# Social costs of icatibant self-administration vs. health professional-administration in the treatment of hereditary angioedema in Spain

**DOI:** 10.1186/2191-1991-3-2

**Published:** 2013-02-12

**Authors:** Antonio J Blasco, Pablo Lázaro, Teresa Caballero, Mar Guilarte

**Affiliations:** 1 Advanced Techniques in Health Services Research (TAISS), Madrid, Spain; 2 Allergy Service, Hospital La Paz Health Research Center (IdiPaz), Biomedical Research Network on Rare Diseases-U754 (CIBERER), Madrid, Spain; 3 Allergy Section, Department of Internal Medicine, Hospital Universitario Vall d´Hebron, Universitat Autònoma de Barcelona, Barcelona, Spain

**Keywords:** C1-INH, Hereditary angioedema, Economic evaluation, Icatibant

## Abstract

**Background:**

Icatibant is the only subcutaneous treatment for acute Type I and Type II hereditary angioedema with C1-esterase inhibitor deficiency (HAE-C1-INH) licensed for self-administration in Europe.

**Aim:**

To compare the economic impact of two icatibant administration strategies: health professional-administration only (strategy 1) versus including the patient self-administration option (strategy 2).

**Methods:**

Economic evaluation model based on the building of a decision tree. Both strategies are assumed to have equivalent effectiveness. The payer (Spanish National Health System) and the social perspectives were considered. All relevant cost-generating factors were taken into account. The time horizon was one year. Sources of information included scientific evidence, official data and experts’ opinion. A deterministic sensitivity analysis was carried out to quantify the underlying uncertainty in the model.

**Results:**

From the social perspective, which considers both direct (health care costs) and indirect costs (productivity losses), strategy 2 would result into average savings of €121.30 per acute attack compared to strategy 1. For Spain, this would achieve in an annual savings of €551,371. The reduction in direct costs accounts for 74% of the savings and lower indirect costs account for the remaining 26%. Savings per acute attack may range from €79.50 to €169.80; accordingly, the annual savings in Spain may vary between €90,319 and €2,315,360.

**Conclusion:**

Costs related to the management of acute HAE attacks with C1 inhibitor deficiency may be substantially reduced through interventions targeting home treatment by training patients to self-administer icatibant.

## Background

Hereditary angioedema with C1-esterase inhibitor deficiency (HAE-C1-INH) is an autosomal dominant hereditary disease caused by deficiency or dysfunction of the protein C1-esterase inhibitor (C1-INH) [1,2]. The decrease in C1-INH activity may increase plasma concentration of bradykinin, the key mediator of HAE-C1-INH symptoms. Patients with this condition experience recurrent episodes of oedema in subcutaneous tissue or submucosa. Areas affected by this swelling include upper respiratory tract, face, limbs, genitals, and digestive tract [1,2]. Laryngopharyngeal oedema may be life-threatening due to upper respiratory tract obstruction [3,4].

Icatibant acetate (Firazyr^®^, Shire HGT), a selective bradykinin B2 receptor competitive antagonist, is among the therapies available for acute HAE-C1-INH attacks [5,6]. Recommended dosage to treat HAE-C1-INH episodes is a 30 mg subcutaneous injection, preferably in the abdomen [7]. A recent clinical study, named “Evaluation of the Safety of Self-Administration with Icatibant (EASSI)”, evaluated the safety, tolerability, convenience, and effectiveness of Firazyr^®^ self-administration [8]. Based on the study’s favourable results, the European Medicines Agency (EMA) has recently approved Firazyr^®^ self-administration for appropriately trained patients in the drug’s self-administration technique [9]. Icatibant is the only subcutaneous treatment for acute Type I and Type II HAE-C1-INH licensed for self-administration in Europe.

Patient’s self-administration of icatibant may reduce healthcare costs and may improve the drug’s effectiveness due to the accessibility of the treatment. Additionally, by reducing the need of health centres or emergency services visits, the patient would miss fewer hours of work, study, or leisure time.

To our knowledge, there is a lack of evaluation studies comparing icatibant administration methods. This study was designed to fill that gap and compare the economic costs of two strategies to manage acute HAE-C1-INH attacks in Spain. The first strategy assumes that only healthcare professionals administer the drug (health professional-only administration) and the second strategy contemplates that, in addition to visiting a health professional, patients have the option of self-administering the drug (self-administration).

## Methods

### Design

This economic evaluation study was developed by building a deterministic decision tree model with sensitivity analysis. The theoretical model compares the costs of health professional-only administration (strategy 1) vs. the costs of self-administration option (strategy 2). Since the model assumes that both strategies are equally effective [8,10,11], this is a cost-minimization study. Analyses were performed from two different perspectives: The payer (Spanish National Health System) perspective which considers direct costs only; and the social perspective which considers both direct and indirect costs. Indirect costs are defined as those incurred from labour productivity losses (lost working hours). The time horizon is one year.

### Model building

**Figure 1:**
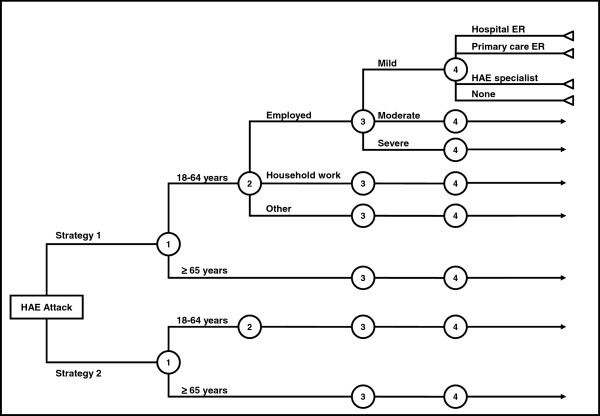
**Simplified model of decision tree. **Icatibant administration: Strategy 1= Health professional-administration only; Strategy 2= Self-administration also available. HAE: Hereditary angioedema. ER: Emergency room.

The model was built in two phases: 1) Decision tree structure; and 2) Values assignation (probabilities and possible values for cost variables). For building the decision tree, the sequence of events was established, and the cost-generating variables and the categories for each variable for each of the two strategies were defined (Table 1). Variables were defined as follows: Attack severity: mild (discomfort that does not disrupt regular daily activities); moderate (discomfort that reduces or impacts regular daily activities); and severe (symptoms preventing work or daily activities) [1]. Episode duration is the time between the onset of symptoms and their complete resolution. Length of stay is the time patients spend in a healthcare facility. Labour force participation categories are: Employed, Unpaid household work and Other (unemployed, students, retired or early retirees due to permanent disability, recipients of pensions other than retirement or early retirement, volunteers in social work, charity organizations, and other [12]). Since icatibant is not approved for younger than 18 years, the age category “under 18” was not considered. The final product was a decision tree structure applicable to HAE episode management for the two treatment strategies. Figure 1 shows a simplified model of the decision tree. During the second phase, the model content was completed assigning probabilities to each of the variables’ categories, and allocating values to the model’s cost variables.

**Table 1:** Variables included in the model

	**Value**		
**Variable**	**Average**	**Minimum**	**Maximum**
**Age (number of residents)**			
18-64 years	29,963,795		
≥ 65 years	7,914,361		
**Work status (%)**			
Employed	59.88%		
Unpaid household work	8.78%		
Other	31.34%		
**HAE Prevalence per 100,000 persons**	2.00	1.00	4.00
**Number of episodes per year**	6.00	3.00	9.00
**Severity (%)**			
Mild	35.00%		
Moderate	45.00%		
Severe	20.00%		
**Episode duration with strategy 1 (hours)**			
Mild	15.00	13.00	18.00
Moderate	10.00	8.00	12.00
Severe	17.00	14.00	21.00
**Reduction of episode duration with strategy 2 (%)**			
Mild	50.00%		
Moderate	60.00%		
Severe	70.00%		
**Episode duration with strategy 2 (hours)**			
Mild	7.50	6.50	9.00
Moderate	4.00	3.20	4.80
Severe	5.10	4.20	6.30
**With strategy 1, a mild episode results in (%)**			
Hospital emergency room visit	2.00%	1.00%	3.00%
Primary care emergency room visit	3.00%	2.00%	4.00%
HAE specialist office visit	2.00%	1.00%	3.00%
No emergency visit	93.00%	96.00%	90.00%
**With strategy 1, a moderate episode results in (%)**			
Hospital emergency room visit	70.00%	60.00%	80.00%
Primary care emergency room visit	5.00%	3.00%	7.00%
HAE specialist office visit	10.00%	7.00%	12.00%
No emergency visit	15.00%	30.00%	1.00%
**With strategy 1, a severe episode results in (%)**			
Hospital emergency room visit	87.00%	85.00%	89.00%
Primary care emergency room visit	2.00%	1.00%	3.00%
HAE specialist office visit	9.00%	6.00%	8.00%
No emergency visit	2.00%	8.00%	0.00%
**With strategy 2, a mild episode results in (%)**			
Hospital emergency room visit	0.00%	0.00%	0.00%
Primary care emergency room visit	0.00%	0.00%	0.00%
HAE specialist office visit	0.00%	0.00%	0.00%
No emergency visit	100.00%	100.00%	100.00%
**With strategy 2, a moderate episode results in (%)**			
Hospital emergency room visit	10.00%	7.00%	12.00%
Primary care emergency room visit	1.00%	0.00%	2.00%
HAE specialist office visit	3.00%	1.00%	5.00%
No emergency visit	86.00%	92.00%	81.00%
**With strategy 2, a severe episode results in (%)**			
Hospital emergency room visit	15.00%	12.00%	18.00%
Primary care emergency room visit	1.00%	0.00%	2.00%
HAE specialist office visit	4.00%	2.00%	6.00%
No emergency visit	80.00%	86.00%	74.00%
**Length of stay in hospital emergency room (hours)**			
Mild	4.0	3.0	6.0
Moderate	7.0	5.0	9.0
Severe	16.0	12.0	20.0
**Length of stay in primary care emergency room (hours)**			
Mild	2.0	1.0	3.0
Moderate	4.0	3.0	5.0
Severe	6.0	4.0	8.0
**Length of stay at the HAE specialist (hours)**			
Mild	1.5	1.0	2.0
Moderate	2.0	1.0	3.0
Severe	5.0	4.0	6.0
**Round trip distance to healthcare facility (Km)**			
To hospital	30.00	20.00	40.00
To primary care centre	4.00	2.00	6.00
**Round trip travel time (minutes)**			
To hospital	45.00	30.00	60.00
To primary care centre	15.00	10.00	20.00
**Timing of episodes among gainfully employed (%)**			
Work hours	21.92%		
Non work hours	78.08%		
**Timing of episodes among unpaid homemakers (%)**			
Work hours	30.59%		
Non work hours	69.41%		
**% episode duration during which unable to work**			
Mild	0.00%	0.0%	0.0%
Moderate	40.00%	30.0%	50.0%
Severe	80.00%	70.0%	90.0%
**Probability (%) patient comes with a caretaker (by age group and episode severity)**			
18-64 years/mild	50.0%	40.0%	60.0%
18-64 years/moderate	60.0%	50.0%	70.0%
18-64 years/severe	95.0%	90.0%	100.0%
≥ 65 years/mild	70.0%	60.0%	80.0%
≥ 65 years/moderate	80.0%	70.0%	95.0%
≥ 65 years/severe	95.0%	90.0%	100.0%
**% of episode duration patient requires caretaker (by age group and episode severity)**			
18-64 years/mild	0.0%	0.0%	0.0%
18-64 years/moderate	20.0%	15.0%	30.0%
18-64 years/severe	70.0%	60.0%	80.0%
≥ 65 years/mild	0.0%	0.0%	0.0%
≥ 65 years/moderate	40.0%	30.0%	50.0%
≥ 65 years/severe	80.0%	70.0%	90.0%
**Icatibant syringes per episode**			
Mild	1.00	0.90	1.10
Moderate	1.10	1.00	1.20
Severe	1.10	1.00	1.30
**With strategy 1, during a severe episode the probability (%) of:**			
Death	0.01%	0.005%	0.015%
Tracheotomy	0.20%	0.10%	0.30%
Hospital admission	0.40%	0.30%	0.50%
**Length of admission for severe episode (days):**			
General ward	2.00	1.00	3.00
ICU	2.00	1.00	3.00
**With strategy 2, during a severe episode the probability (%) of:**			
Death	0.0010%	0.0005%	0.0015%
Tracheotomy	0.0200%	0.0100%	0.0300%
Hospital admission	0.1000%	0.0500%	0.1500%

Icatibant administration: Strategy 1= Health professional-administration only; Strategy 2= Self-administration also available.

### Probability assignment

Probabilities were assigned based on the best available scientific evidence. In some cases, data were not available or were available for other populations and, thus, the experts (TC, MG) deemed them inappropriate for modelling Spanish events. In these cases, the assignment of probabilities was informed by experts’ opinions.

Official data sources were used for the age variable (Spain’s population as of April 1st, 2011 [13]) and for the labour participation variable (Labour Force Survey, First Quarter, 2011 [12]).

Experts’ opinions informed the parameterization of the remaining variables by assigning estimates of average, maximum, and minimum values for each variable. The experts parameterized the variables anonymously in two separate rounds with no interaction during the first round. The mean of these estimates became the synthesis estimators and during the second round the final values were allocated by consensus.

### Cost allocation

The payer’s perspective accounts only for direct medical costs (treatment, hospital emergency room visit, primary care emergency room visit, HAE specialist visit, hospital admission, ICU admission, and tracheotomy) and non-medical costs (transportation).

Because the healthcare system in Spain is governed by the autonomous communities (AC) (political geographic divisions), there are not national prices. Thus, a resource unit cost based on the official AC lists of resource unit costs [14-31] was estimated. This cost was calculated as the average price for healthcare services rendered by the AC Departments of Health. Treatment cost was the pharmaceutical laboratory sale’s price (LSP) plus the value added tax (VAT).

Transportation costs were estimated using data from the *Centro de Estudios y Experimentación de Obras Públicas* (CEDEX) (Center for Public Works Studies and Experimentation) [32]. CEDEX provides estimates of the cost per kilometre of private transportation taking into account the investment, maintenance, vehicle repairs, tires change, insurance policy, motor vehicle tax, gasoline, parking, fines, and tolls.

The social perspective accounts for both direct and indirect costs. Indirect costs are defined as patient’s and caretaker’s working hours lost per episode plus working hours lost in case of patient’s death. Leisure time lost was not included in the calculation. For employed workers, labour cost was estimated using the human capital method, based on the cost of the working hour reported in the Quarterly Survey of Labour Costs during the Fourth Quarter of 2010 [33]. For unpaid work (household work) the substitution cost method was used.

The cost of lost labour due to the patient’s death was estimated multiplying the years the patient would no longer contribute to the national wealth (contributing years lost) times the per capita gross domestic product (GDP). The per capita GDP was calculated dividing the national 2009 GDP (last available datum) [34] by the Spanish population count as of July 1st, 2009 [13]. To calculate contributing years lost, it was assumed that, in average, patients under 65 years-old die at 41.5 years of age. For those 65 and over, death occurs half way between 65 and life expectancy at 65. The amount of contributing years lost is the difference between life expectancy [35] and age at death.

The product of this phase was the final model: a decision tree loaded with the probability values and the costs associated with each path.

### Sensitivity analysis

A sensitivity analysis was performed to evaluate the underlying uncertainty dependent on the variability in the experts’ estimators and the resources unit costs. Three scenarios were built under this analysis: base case scenario; most favourable (for strategy 2 vs. strategy 1); and least favourable (for strategy 2 vs. strategy 1). Unit costs for resources were allowed a potential variability of ± 5%.

Base case scenario employs the average unit costs and the average experts’ estimators. The most favourable scenario uses the maximum unit costs and maximum experts’ estimators, except for the following variables in strategy 2: Facility in which episode is treated, probability of death given a severe episode, probability of tracheotomy given a severe episode, and probability of admission given a severe episode for which the minimum estimators were entered. For the least favourable scenario the minimum unit costs and minimum experts’ estimators were considered, except for the variables mentioned above in strategy 2 for which the maximum estimators were entered.

## Results

Table 1 shows the values assigned to the variables in the model. Table 2 shows unit costs of the resources included in the model.

**Table 2:** Unit costs of resources used in 2011

**Variable**	**Cost (€)**	**Unit**
**Direct costs**		
***Medical***		
Icatibant	1,762.80	syringe 30 mg
Hospital emergency	169.73	visit
Primary care emergency services	100.76	visit
HAE specialist	131.67	visit
General ward admission	443.61	day
ICU admission	1,115.61	day
Tracheotomy	464.66	tracheotomy
***Non-medical***		
Transportation	0.45	km
**Indirect costs**		
Employee loss labour	17.27	hour
Homemaker loss labour	10.00	hour
Death among 18–64 year-olds	921,984	death
Death among ≥ 65 year-olds	232,446	death

In the base case scenario, HAE-C1-INH prevalence is 2 per 100,000 persons and each patient suffers an average of 6 acute attacks per year. Consequently, it is estimated that in Spain there would be 758 HAE-C1-INH patients experiencing a total of 4,545 acute attacks per year. Using the social perspective, the average cost of managing one of these episodes with strategy 1 (health professional-administration) would be €1,315.14 versus €1,193.84 with strategy 2 (self-administration option). This translates into an average savings of €121.30 (9.2% cost reduction) per episode with strategy 2, representing an annual saving of €551,371 nationwide. A reduction in direct costs would account for 74% of the savings and a decrease in indirect costs would make up the remaining 26% of the money saved. With the payer perspective, the average savings per episode would be €89.8 (7%), which would result into an annual saving of €408,157 nationwide. The decrease in healthcare services use (emergency services and visits to specialists) accounts for the bulk of the savings (Table 3).

**Table 3:** Cost of managing HAE episodes with icatibant (Euros)

	**Per episode**			**Nationwide per year**			
**Variable**	**Strategy 1**	**Strategy 2**	**Savings**	**Strategy 1**	**Strategy 2**	**Savings**	**Savings (%)**
**Direct costs**	**1,272.42**	**1,182.62**	**89.80**	**5,783,612**	**5,375,455**	**408,157**	**7.06**
***Medical***	***1,264.71***	***1,181.31***	***83.40***	***5,748,578***	***5,369,480***	***379,098***	***6.59***
Icatibant	1,164.95	1,164.95	0.00	5,295,122	5,295,122	0	0.00
Visits*	97.13	16.22	80.92	441,498	73,706	367,792	83.31
Admissions	2.44	0.12	2.32	11,113	567	10,546	94.90
Tracheotomy	0.19	0.02	0.17	845	84	760	90.00
***Non-medical***	***7.71***	***1.31***	***6.39***	***35,034***	***5,975***	***29,059***	***82.95***
Transportation	7.71	1.31	6.39	35,034	5,975	29,059	82.95
**Indirect costs**	**42.73**	**11.22**	**31.51**	**194,220**	**51,006**	**143,214**	**73.74**
Caretaker	13.54	4.81	8.73	61,535	21,844	39,691	64.50
Labour loss	13.63	4.86	8.77	61,967	22,090	39,877	64.35
Death	15.56	1.56	14.00	70,718	7,072	63,646	90.00
**Social cost**	**1,315.14**	**1,193.84**	**121.30**	**5,977,832**	**5,426,461**	**551,371**	**9.22**

Strategy 1= Health professional-administration only; Strategy 2= Self-administration also available.

*Includes visits to hospital emergency rooms, primary care centres emergency rooms, and to HAE specialist offices.

The number of episodes and the average treatment cost (with the social perspective), according to patient’s age and severity of the attack is showed in Table 4. The more severe the episode, the greater the savings are. Savings are also greater in patients under 65 than in those 65 and over, due to the greater reduction in indirect costs.

**Table 4:** Number of episodes and average cost (Euros) based on the social perspective, according to age group and episode severity

			**Cost (€)**			
**Age**	**Severity**	**Episodes (N)**	**Strategy 1**	**Strategy 2**	**Savings (€)**	**Savings (%)**
18-64 years	Mild	1,258	133.8	123.4	10.43	7.80
	Moderate	1,618	1,826.3	1,681.6	144.76	7.93
	Severe	719	2,262.2	1,976.2	285.99	12.64
	Total	3,596	1,321.1	1,195.1	125.99	9.54
≥ 65 years	Mild	332	133.4	123.4	10.01	7.51
	Moderate	427	1,812.3	1,677.7	134.52	7.42
	Severe	190	2,151.5	1,953.9	197.59	9.18
	Total	950	1,292.5	1,189.0	103.56	8.01
TOTAL		4,545	1,315.1	1,193.8	121.30	9.22

Strategy 1= Health professional-administration only; Strategy 2= Self-administration also available.

### Sensitivity analysis

In the most favourable scenario for strategy 2, HAE-C1-INH prevalence is 4 per 100,000 persons and each patient experiences an average of 9 acute attacks per year. This would add up to 1,515 patients suffering a total of 13,636 acute attacks per year. Under the social perspective, the average cost of managing an episode would be €1,664.21 with strategy 1 and €1,494.41 with strategy 2. Therefore, strategy 2 (self-administration option) would save an average of €169.80 (10.2%) per episode (Table 5) which would translate into an annual saving of €2,315,360 nationwide. The reduction in direct costs would account for 70% of the savings and a decrease in indirect costs would explain the remaining 30% (Table 6). With the payer perspective, the average savings would be €119.21 (7.5%) which represent an annual saving of €1,625,616 nationwide.

**Table 5:** Average cost of managing HAE episodes with icatibant (Euros)

	**Most favourable scenario**				**Least favourable scenario**			
**Variable**	**Strategy 1 (€)**	**Strategy 2 (€)**	**Savings (€)**	**Savings (%)**	**Strategy 1 (€)**	**Strategy 2 (€)**	**Savings (€)**	**Savings (%)**
**Direct costs**	**1,600.16**	**1,480.94**	**119.21**	**7.45**	**986.69**	**923.07**	**63.62**	**6.45**
***Medical***	***1,588.12***	***1,479.73***	***108.39***	***6.82***	***982.48***	***922.00***	***60.48***	***6.16***
Icatibant	1,468.59	1,468.59	0.00	0.00	901.85	901.85	0.00	0.00
Visits*	114.33	11.07	103.26	90.32	79.54	20.07	59.47	74.77
Admissions	4.91	0.07	4.84	98.60	0.82	0.05	0.77	93.78
Tracheotomy	0.29	0.01	0.28	96.67	0.26	0.03	0.24	90.00
***Non-medical***	***12.03***	***1.21***	***10.82***	***89.95***	***4.21***	***1.08***	***3.14***	***74.47***
Transportation	12.03	1.21	10.82	89.95	4.21	1.08	3.14	74.47
**Indirect costs**	**64.05**	**13.47**	**50.58**	**78.97**	**25.21**	**9.34**	**15.87**	**62.95**
Caretaker	20.56	6.47	14.09	68.53	8.62	3.49	5,14	59.55
Labor loss	18.98	6.18	12.80	67.45	9.19	3.63	5.56	60.46
Death	24.50	0.82	23.69	96.67	7.39	2.22	5.17	70.00
**Social costs**	**1,664.21**	**1,494.41**	**169.80**	**10.20**	**1,011.89**	**932.41**	**79.48**	**7.85**

Strategy 1= Health professional-administration only; Strategy 2= Self-administration also available.

*Includes visits to hospital emergency rooms, primary care centres emergency rooms, and to HAE specialist offices.

Sensitivity analysis.

**Table 6:** Nationwide annual cost of managing HAE episodes with icatibant (Euros)

	**Most favourable scenario**				**Least favourable scenario**			
**Variable**	**Strategy 1 (€)**	**Strategy 2 (€)**	**Savings (€)**	**Savings (%)**	**Strategy 1 (€)**	**Strategy 2 (€)**	**Savings (€)**	**Savings (%)**
**Direct costs**	**21,819,956**	**20,194,340**	**1,625,616**	**7.45**	**1,121,218**	**1,048,929**	**72,289**	**6.45**
***Medical***	***21,655,858***	***20,177,846***	***1,478,012***	***6.82***	***1,116,431***	***1,047,707***	***68,723***	***6.16***
Icatibant	20,025,875	20,025,875	0	0.00	1,024,811	1,024,811	0	0.00
Visits*	1,559,016	150,900	1,408,116	90.32	90,390	22,808	67,581	74.77
Admissions	66,974	938	66,037	98.60	929	58	871	93.78
Tracheotomy	3,992	133	3,859	96.67	301	30	271	90.00
***Non-medical***	***164,099***	***16,494***	***147,604***	***89.95***	***4,787***	***1,222***	***3,565***	***74.47***
Transportation	164,099	16,494	147,604	89.95	4,787	1,222	3,565	74.47
**Indirect costs**	**873,371**	**183,627**	**689,743**	**78.97**	**28,643**	**10,613**	**18,030**	**62.95**
Caretaker	280,398	88,246	192,152	68.53	9,800	3,964	5,837	59.55
Labor loss	258,830	84,243	174,587	67.45	10,445	4,130	6,315	60.46
Death	334,142	11,138	323,004	96.67	8,398	2,519	5,878	70.00
**Social costs**	**22,693,327**	**20,377,967**	**2,315,360**	**10.20**	**1,149,861**	**1,059,542**	**90,319**	**7.85**

Strategy 1= Health professional-administration only; Strategy 2= Self-administration also available.

*Includes visits to hospital emergency rooms, primary care centres emergency rooms, and to HAE specialist offices.

Sensitivity analysis.

In the least favourable scenario for strategy 2, HAE-C1-INH prevalence is 1 per 100,000 persons and each patient suffers an average of 3 acute attacks per year. That is, there would be 379 patients experiencing a total of 1,136 acute attacks per year. Under the social perspective, the average cost of managing an episode would be €1,011.89 with strategy 1 and €932.41 with strategy 2. Thus, strategy 2 yields an average savings of €79.48 per episode (7.9%) (Table 5). This leads to an annual saving of €90,319 nationwide. Direct costs reduction would account for 80% of the savings and a decrease in indirect costs would explain the remaining 20% (Table 6). Using the payer perspective, the average saving per episode would be €63.62 (6.5%) which would represent an annual saving of €72,289 nationwide.

Table 7 shows the number of episodes and the average treatment cost (with the social perspective) according to patient’s age and severity of the attack, for the most and least favourable scenarios.

**Table 7:** Number of episodes and average cost (Euros) with the social perspective, according to age group and episode severity

		**Most favourable scenario**					**Least favourable scenario**				
**Age**	**Severity**	**Episodes (N)**	**Strategy 1 (€)**	**Strategy 2 (€)**	**Savings (€)**	**Savings (%)**	**Episodes (N)**	**Strategy 1 (€)**	**Strategy 2 (€)**	**Savings (€)**	**Savings (%)**
18-64 years	Mild	3,775	210.7	193.9	16.8	7.98	315	68.7	63.5	5.2	7.59
	Moderate	4,854	2,325.1	2,122.8	202.3	8.70	405	1,365.6	1,269.4	96.2	7.04
	Severe	2,157	2,764.7	2,363.6	401.1	14.51	180	1,883.9	1,700.6	183.3	9.73
	Total	10,787	1,673.0	1,495.9	177.2	10.59	899	1,015.3	933.6	81.8	8.05
≥ 65 years	Mild	997	209.8	193.9	15.9	7.58	83	68.5	63.5	5.1	7.40
	Moderate	1,282	2,304.9	2,117.6	187.3	8.13	107	1,357.2	1,266.7	90.6	6.67
	Severe	570	2,601.0	2,340.8	260.3	10.01	47	1,820.4	1,678.9	141.6	7.78
	Total	2,849	1,630.9	1,489.0	141.9	8.70	237	998.8	928.0	70.8	7.09
TOTAL		13,636	1,664.2	1,494.4	169.8	10.20	1,136	1,011.9	932.4	79.5	7.85

Strategy 1= Health professional-administration only; Strategy 2= Self-administration also available.

Sensitivity Analysis.

In summary, the cost comparison between strategy 2 and 1, shows that with the social perspective, savings per episode would be of €121.3 that could range from €79.5 to €169.8. This equals to save the 9.2% of the costs, which could range between 7.9% and 10.2%. Annual cost reduction in Spain would be of €551,371 ranging from €90,319 to €2,315,360.

With the payer perspective, savings per episode would be €89.80 that could range from €63.60 to €119.20. This equals to a savings of 7% which could range from 6.5% to 7.5%. Annual cost reduction in Spain would be €408,157 ranging between €72,289 and €1,625,616.

## Discussion

According to this study’s findings, the possibility of patients self-administering icatibant to control acute HAE-C1-INH attacks brings about substantial reduction in both direct and indirect costs, resulting in savings for the National Health System and for the society as a whole. To our knowledge and based on our literature search, this is the first study to date that has evaluated the economic costs involved in self-administration.

One of the strengths of the current study is that it comprises all relevant variables impacting the cost of managing acute HAE-C1-INH attacks, including the indirect costs related to patients’ and caretakers’ loss of labour hours. Further, these indirect costs also account for the years of life lost due to the unfortunate premature deaths which, though uncommon, do occur in this patient population.

The main study limitation is the scant scientific evidence available on the study variables. In the absence of scientific evidence, the next best alternative to fill this information gap is the experts’ opinion. Although the panel consisted of only two experts, they are both renowned opinion leaders on this disease with ample experience in the management of HAE-C1-INH in Spain. Another possible limitation of the study is that the costs of training patients to self-administer the treatment were not taken into account. However, these costs would be irrelevant, since, in practice, the patients receive the training in the regular visits to the specialist. Finally, the model assumes neither underuse nor overuse of icatibant in the two compared strategies. However, it would be possible that in some settings, overuse or underuse may occur.

The alternative of self-administration of icatibant opens the possibility of early treatment of acute attacks at the first symptoms which may decrease attack severity [36,37]. For instance, potentially severe attacks may turn into mild attacks thanks to early treatment which is likely to lead to even greater savings than those estimated in this study.

In addition, the unpredictability of the timing, frequency, and severity of acute HAE-C1-INH attacks generate a substantial amount of stress in patients. Having the means to control an acute attack quickly and effectively may reduce that stress. This piece of mind compounds another unquantifiable benefit such as the patient’s quality of life improvement derived from the reduction of previous restrictions in daily activities as well as work and school absenteeism [37,38].

## Conclusions

Both the clinical aspects of HAE-C1-INH and, now, the economic aspects revealed in this study, strongly suggest the need for targeted interventions. These interventions would address home availability of the treatment specific to acute attacks as proposed by national and international consensus [38-41] and HAE-C1-INH patients’ training on the proper technique of icatibant self-administration.

## Abbreviations

AC: Autonomous communities; C1-INH: C1-esterase inhibitor; CEDEX: *Centro de Estudios y Experimentación de Obras Públicas *(Center for Public Works Studies and Experimentation); GDP: Gross domestic product; HAE-C1-INH: Angioedema with C1-esterase inhibitor deficiency.

## Competing interests

This study was supported by Shire Pharmaceuticals Ibérica. TC has received sponsorship for educational purposes, has been paid for providing consultancy services, and has taken part in clinical trials sponsored by Jerini AG/Shire, CSL Behring, Dyax Corp, Pharming NV, and ViroPharma Incorporated. MC has received sponsorship for educational purposes and has been paid for providing consultancy services from CSL Behring, Jerini AG/Shire, and ViroPharma Incorporated; and has taken part in clinical trials sponsored by Jerini AG/Shire, Pharming NV and ViroPharma Incorporated. AJB and PL work as researchers in TAISS; TAISS has received funding from Shire for developing the project.

## Authors’ contributions

AJB did substantial contributions to the project conception and design, participated in the data acquisition and in the analysis and interpretation of data, prepared the draft of the article and approved the final version. PL did substantial contributions to conception and design, participated in the acquisition of data and in the analysis and interpretation of data, revised the manuscript critically for important intellectual content and approved the final version. TC did substantial contributions to conception and design, participated in the acquisition of data and in the interpretation of data, revised the manuscript critically for important intellectual content and approved the final version. MG did substantial contributions to conception and design, participated in the acquisition of data and in the interpretation of data, revised the manuscript critically for important intellectual content and approved the final version. All authors read and approved the final manuscript.
